# Harmonizing patient health data into the OMOP common data model: a case study using tuberculosis data from Douala General Hospital, Cameroon

**DOI:** 10.3389/fdgth.2026.1828475

**Published:** 2026-08-03

**Authors:** Brenda Mbouamba Yankam, Fankoua Tchaptchet Luc Baudoin, François Anicet Onana Akoa, Pauline Andeso, Steve Bicko Cygu, Samuel Iddi, Michael Ochola, Tathagata Bhattacharjee, Mbele Onana Charles Lebon, Miranda Barasa, Agnes Kiragga, Bertrand Hugo Mbatchou Ngahane

**Affiliations:** 1 Data Science Without Borders Project, Douala General Hospital, Douala, Cameroon; 2 Faculty of Mathematics, University of Bochum, Bochum, Germany; 3 School of Health Sciences, Catholic University of Central Africa, Yaounde, Cameroon; 4 African Population and Health Research Center (APHRC), Nairobi, Kenya; 5 Department of Statistics, University of Ghana, Accra, Ghana; 6 Department of Population Health, Faculty of Epidemiology and Population Health, London School of Hygiene and Tropical Medicine, University of London, London, United Kingdom; 7 Internal Medicine Department, Douala General Hospital, Douala, Cameroon; 8 Infectious Diseases Institute, College of Health Sciences, Makerere University, Kampala, Uganda; 9 Faculty of Medicine and Pharmaceutical Sciences, University of Douala, Douala, Cameroon

**Keywords:** data harmonization, ETL, interoperability, OHDSI, OMOP CDM, tuberculosis

## Abstract

**Introduction:**

Douala General Hospital is a tertiary healthcare facility in Cameroon that serves thousands of patients annually. Despite maintaining extensive patient records with potential for public health research, most data remain paper-based, limiting accessibility, reuse, and interoperability. In departments such as pulmonology, patient information is stored in heterogeneous, non-standardized formats, constraining its use for clinical research, care management, and decision-making. To address these challenges, we implemented a complete Extract, Transform, and Load (ETL) pipeline aligned with the Observational Medical Outcomes Partnership (OMOP) Common Data Model (CDM) Version 5.4.

**Objective:**

This study aimed to harmonise and integrate tuberculosis (TB) patient data into the OMOP CDM to improve data quality, interoperability, and reusability for research and clinical monitoring.

**Methods:**

A retrospective TB dataset covering 2009–2022 was digitized from paper records and included 2,426 patient records with 82 variables. Data profiling was performed using WhiteRabbit, mapping with Rabbit-in-a-Hat, and vocabulary alignment using Usagi and ATHENA. Transformed data were loaded into a PostgreSQL OMOP CDM database. Data quality was evaluated using Achilles and the Data Quality Dashboard (DQD), while ATLAS supported visualization and analytical exploration.

**Results:**

Of 2,374 DQD checks executed, 2,354 passed, and 42 failed, yielding a 99% overall pass rate. Many checks that passed were not applicable to this TB-specific dataset, and all applicable checks demonstrated high data quality after iterative ETL refinement. Most issues were minor and resolved during refinement. Demographically, 55.6% of patients were male, with ages ranging from 15 to 95 years, predominantly between 30 and 40 years. Common conditions included extrapulmonary TB, fever, cough, and chest pain. Most patients contributed one condition and one observation record, reflecting the dataset's cross-sectional nature.

**Conclusion:**

Harmonising TB data into the OMOP CDM was feasible and successful, demonstrating that standardized research datasets can be generated in resource-limited settings. This approach offers a scalable model for broader health data standardisation efforts in Cameroon and similar contexts.

## 1 Introduction

Tuberculosis (TB) remains a significant global health challenge, particularly in Sub-Saharan Africa (SSA), where the disease burden is substantial, and healthcare resources are often limited (1, 2). Cameroon remains a high-TB-burden country and faces ongoing challenges in TB surveillance, treatment monitoring, and outcomes assessment (3, 4). Douala General Hospital (DGH), one of the leading tertiary healthcare institutions in Cameroon's economic capital, manages a considerable volume of TB cases annually (5). However, like many healthcare facilities in resource-limited settings, the hospital's TB data exists in heterogeneous formats, utilising local coding systems and non-standardized terminology, which impede comprehensive data analysis and international research collaboration.

These data challenges at DGH reflect broader issues affecting healthcare systems globally. The increasing availability of electronic health records (EHRs) and clinical databases has created new opportunities for healthcare research, quality improvement, and evidence-based decision-making. However, the heterogeneity of healthcare data systems, varying terminologies, and diverse data structures across institutions pose major challenges to data interoperability and the secondary use of clinical data. This fragmentation limits the ability to conduct multi-centre research, implement comparative effectiveness studies, and generate real-world evidence to support clinical practice and public health decision-making (6). It is worth emphasising, however, that the availability of EHR systems does not inherently guarantee well-structured or analysis-ready data.

To address these challenges, Common Data Models (CDMs) have emerged as robust solutions for standardising healthcare data from diverse sources (7). Among these, the Observational Medical Outcomes Partnership (OMOP) Common Data Model (CDM) has gained substantial adoption due to its comprehensive design, rich vocabulary ecosystem, and active community support through the Observational Health Data Sciences and Informatics (OHDSI) collaborative network (7, 8). The OMOP CDM provides a standardized structure that enables systematic organization of diverse healthcare data while maintaining semantic consistency through standardized vocabularies and concept identifiers (8, 9).

The effectiveness of OMOP CDM as a standardisation solution has been validated across numerous healthcare settings and disease areas. Recent implementations of the OMOP CDM across various healthcare contexts have demonstrated its versatility and effectiveness (10–13). Studies have shown the successful transformation of EHR data, disease registries, and databases into the OMOP format, enabling large-scale observational research and quality assessments. For instance, Biedermann et al. (14) successfully standardized three pulmonary hypertension databases to the OMOP CDM, demonstrating the model's applicability to specialised disease registries. Similarly, Papez et al. (15) illustrated how the OMOP CDM facilitates the transformation and evaluation of EHR disease phenotyping algorithms, particularly in cardiovascular conditions. The model has also proven valuable in rare disease research, where data harmonisation is particularly challenging due to small patient populations and diverse clinical presentations. Ahmadi et al. (16) provided comprehensive guidance on customising the OMOP CDM for rare diseases, highlighting the flexibility of the framework to accommodate specialised research needs. Furthermore, the OMOP CDM has shown significant usefulness in oncology research, as observed in a scoping review that revealed increasing adoption of OMOP CDM for cancer research using real-world data, emphasising its potential for advancing precision medicine initiatives (9).

However, successful OMOP CDM implementation extends beyond mere data transformation; it encompasses critical considerations of data quality, semantic interoperability, and analytical capabilities. Kahn et al. (17) established a harmonised data quality assessment terminology and framework specifically designed for the secondary use of EHR data, providing foundational principles that guide CDM implementations. Building on these work, Blacketer et al. (18) demonstrated how the Data Quality Dashboard (DQD) can systematically improve data quality across healthcare networks, ensuring that transformed data meet rigorous standards for research and clinical decision support. Moreover, Marteau et al. (11) utilised OHDSI's DQD to enhance a large healthcare system's research data warehouse, identifying and addressing critical data quality issues that could compromise research validity.

Supporting these quality assurance efforts, the technical infrastructure for OMOP CDM implementations has evolved considerably, with various OHDSI tools facilitating different aspects of the transformation process. For instance, Quiroz et al. (19) developed a comprehensive Extract, Transform, Load (ETL) framework for converting health databases to OMOP, providing methodological guidance that has informed subsequent implementations worldwide. The versatility of OMOP CDM extends across diverse healthcare contexts, including process mining applications, as explored by Park et al. (12), who demonstrated how standardized data structures enable sophisticated analytical approaches beyond traditional observational research. Moreover, Voss et al. (20) demonstrated the scalability of OMOP CDM implementations, documenting valuable lessons learned from building a standardized international health data network spanning multiple countries and healthcare systems.

### 1.1 Rationale for implementation

The implementation of OMOP CDM for TB data at DGH was undertaken to address several critical needs and objectives. There is a pressing need to standardize TB clinical data to enable meaningful comparisons with international TB registries and research databases, facilitating participation in global TB research programs. Moreover, transforming local TB data into a standardized format is essential to improving data quality, identifying data gaps, conducting cross-country analysis, and establishing reliable, efficient data governance practices that support both clinical care and research activities.

Furthermore, the implementation serves as a proof-of-concept for broader health data harmonization efforts in Cameroon and similar resource-limited settings, where standardized data infrastructure is often lacking (21, 22). The experience gained from this TB-focused implementation provides valuable insights into the challenges and opportunities of OMOP CDM adoption in Cameroon and across SSA healthcare contexts (21), potentially informing future data standardization initiatives across the region.

The challenges encountered when implementing OMOP CDM in specific disease contexts have been well documented in the literature. Frid et al. (23) evaluated OMOP CDM alongside other data models to support data harmonization in a breast cancer use case, highlighting important considerations for disease-specific implementations. Similarly, the implementation of OMOP in healthcare consortium settings, as described by Maier et al. (24), provides valuable lessons on institutional coordination, technical infrastructure requirements, and stakeholder engagement strategies. The feasibility of transforming diverse data sources, including questionnaire data alongside EHRs, has been demonstrated using diabetes datasets, confirming the model's flexibility in accommodating various data types relevant to disease management (25).

This manuscript, therefore, presents a detailed account of the methodology employed to harmonize TB patient data from DGH into the OMOP CDM using OHDSI tools.

## 2 Methodology

### 2.1 Source data

Douala General Hospital is a leading tertiary-level healthcare institution in Cameroon, offering specialized services to a diverse patient population and recording nearly 100,000 outpatient consultations in 2023. The source data, an in-patient TB dataset from DGH initially collected in paper-based form, was digitized that is, systematically converted from paper clinical records into structured electronic data to enable efficient storage, retrieval, and secondary use for analysis and research, and covered the period from 2009 to 2022, comprising 2,426 records and 82 variables, representing a wide range of socio-demographic characteristics, including gender, age, occupation, medical history such as HIV status, hepatitis serology, symptoms such as fever, cough, chest pain, and laboratory parameters including creatinine levels, neutrophils, and eosinophils.

The digitization was performed with careful verification against original files to ensure accuracy, followed by manual review by DGH clinicians to ensure semantic consistency that retains the actual meaning of the data prior to vocabulary mapping. The digitization process itself represented a significant resource investment unique to low and middle-income settings, such as the case of Cameroon: trained data entry personnel manually transcribed paper registers over several months, with cross-checking performed to minimize transcription errors.

**Figure 1:**
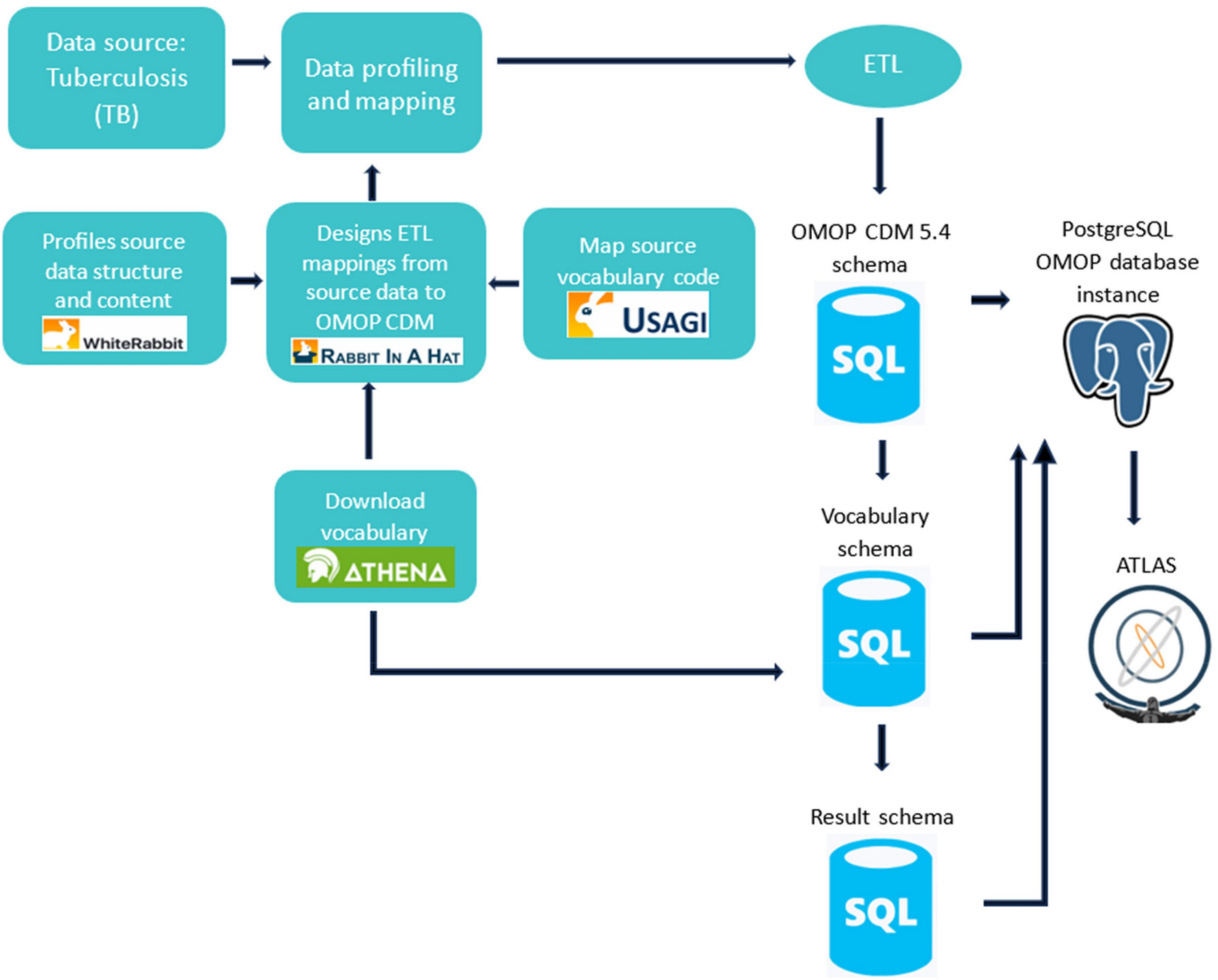
OMOP CDM implementation pipeline.

After digitization and verification of the dataset, the dataset was cleaned in R (v4.5.1), including the removal of duplicates and the resolution of missing or inconsistent values by rechecking patient files, and quality checks were performed before initiating the OMOP CDM implementation steps shown in Figure 1.

A descriptive statistic was performed on the cleaned dataset. Among the 2,426 participants included in the analysis, 237 (9.8%) reported a history of tobacco consumption, 889 (48.0%) reported having a cough, and 467 (25.3%) reported chest pain. The age distribution showed that the largest proportion of participants was aged 21–35 years (35.4%), followed by 36–45 years (25.6%) and 46–55 years (16.3%). History of tobacco consumption was most frequently reported among individuals aged 21–45 years, accounting for over 50% of all tobacco users, while cough and chest pain were also most prevalent in the 21–35 years age group, representing 37.1% and 36.4% of those reporting each symptom, respectively, as presented in Table 1.

**Table 1:** Frequency and percentage of participants with history of tobacco consumption, fever, cough, and chest pain stratified by sociodemographic characteristics.

Sociodemographic characteristics	History of tobacco consumption		Cough		Chest pain		Total
	Yes	No	Yes	No	Yes	No	
Age	*N* = 237	*N* = 2,189	*N* = 889	*N* = 962	*N* = 467	*N* = 1,379	*N* = 2,426
15–20	5 (2.11)	169 (7.72)	74 (8.32)	67 (6.96)	42 (8.99)	97 (7.03)	174 (7.17)
21–35	60 (25.32)	798 (36.46)	330 (37.12)	316 (32.85)	170 (36.40)	476 (34.52)	858 (35.37)
36–45	61 (25.74)	559 (25.54)	206 (23.17)	247 (25.68)	102 (21.84)	351 (25.45)	620 (25.56)
46–55	55 (23.21)	340 (15.53)	142 (15.97)	162 (16.84)	75 (16.06)	227 (16.46)	395 (16.28)
56–65	39 (16.46)	216 (9.87)	92 (10.35)	110 (11.43)	49 (10.49)	152 (11.02)	255 (10.51)
66–75	12 (5.06)	76 (3.47)	28 (3.15)	46 (4.78)	16 (3.43)	58 (4.21)	88 (3.63)
76–85	5 (2.11)	27 (1.23)	16 (1.80)	12 (1.25)	13 (2.78)	15 (1.09)	32 (1.32)
86–95	0 (0.00)	4 (0.18)	1 (0.11)	2 (0.21)	0 (0.00)	3 (0.22)	4 (0.16)
96 and above	0 (0.00)	0 (0.00)	0 (0.00)	0 (0.00)	0 (0.00)	0 (0.00)	0 (0.00)
Sex							
Female	58 (24.47)	1,019 (46.55)	380 (42.74)	414 (43.04)	181 (38.76)	609 (44.16)	1,077 (44.39)
Male	179 (75.53)	1,170 (53.45)	509 (57.26)	548 (56.96)	286 (61.24)	770 (55.84)	1,349 (55.61)
Single	24 (10.13)	303 (13.84)	186 (20.92)	141 (14.66)	96 (20.56)	231 (16.75)	327 (13.48)
Marital Status							
Married	38 (16.03)	284 (12.97)	186 (20.92)	136 (14.14)	87 (18.63)	235 (17.04)	322 (13.27)
Widow	4 (1.69)	34 (1.55)	20 (2.25)	18 (1.87)	8 (1.71)	30 (2.18)	38 (1.57)
Divorce	1 (0.42)	3 (0.14)	3 (0.34)	1 (0.10)	0 (0.00)	4 (0.29)	4 (0.16)
Occupation							
Employed	167 (70.46)	727 (33.21)	528 (59.39)	336 (34.93)	287 (61.46)	575 (41.70)	894 (36.85)
Student	23 (9.70)	247 (11.28)	171 (19.24)	93 (9.67)	95 (20.34)	167 (12.11)	270 (11.13)
Unemployed	19 (8.02)	223 (10.19)	143 (16.09)	90 (9.36)	62 (13.28)	171 (12.40)	242 (9.98)
Retired	21 (8.86)	45 (2.06)	40 (4.50)	26 (2.70)	20 (4.28)	46 (3.34)	66 (2.72)
**Total**	**237** (**100)**	**2,189 (100)**	**889** (**100)**	**962** (**100)**	**467** (**100)**	**1,379 (100)**	**2,426 (100)**

The bold values indicate the number of observations (records) per category in our dataset.

Female participants accounted for 44.4% of the total sample, whereas male participants accounted for 55.6%. A higher proportion of males reported a history of tobacco consumption (75.5%) compared to females (24.5%), and males also slightly predominated among those reporting cough (57.3%) and chest pain (61.2%).

Regarding marital status, data were available for 691 participants. Among those with a history of tobacco consumption, married individuals represented the largest subgroup (16.0%), followed by single participants (10.1%). Single and married participants contributed similarly to cough reports (20.9% each), while single participants showed a slightly higher proportion of chest pain (20.6%) compared to married participants (18.6%).

For occupation, data were available for 1,472 participants with tobacco history records and 1,427 with cough and chest pain records. Employed individuals accounted for the largest share of tobacco consumption history (70.5%), cough (59.4%), and chest pain (61.5%), followed by students and unemployed participants. Retired individuals consistently represented the smallest occupational group across all three characteristics.

To enable replication of this workflow in similar resource-limited settings, the end-to-end process can be summarized as follows: (1) paper clinical records were manually transcribed by trained personnel into a structured electronic spreadsheet; (2) the digitized data were verified against original files and reviewed by DGH clinicians for semantic accuracy; (3) the dataset was cleaned in R, with duplicates removed and missing values resolved by cross-checking patient files; (4) the cleaned CSV file was profiled using WhiteRabbit, mapped using Rabbit-in-a-Hat, and vocabulary-aligned using Usagi and ATHENA; (5) ETL scripts were developed in SQL to load the transformed data into a PostgreSQL database structured according to OMOP CDM Version 5.4; and (6) data quality was assessed iteratively using Achilles and DQD until all applicable checks passed.

Note. The dataset metadata, not the patient-level data, was subsequently registered in the eLwazi catalogue via REDCap to support discoverability and governance. This metadata is recorded under the DSWB project, named *Tuberculosis Dataset*, registered under Cameroon. The cleaned patient-level dataset was processed directly through the OMOP ETL pipeline described in 2.3.1 to 2.3.7, without routing through REDCap. Access to the metadata for further research can be obtained by contacting the designated person listed in the eLwazi catalogue (https://catalog.elwazi.org/#/datasets).

### 2.2 Overview of the implementation approach

The transformation of TB patient data from DGH into the OMOP CDM followed a systematic approach utilising the OHDSI tools. This approach encompassed six primary phases: source data profiling, conceptual mapping design, terminology mapping to standard vocabularies, database infrastructure setup, data transformation and loading, and quality assessment and validation, as presented in Figure 1.

The source data, a cleaned CSV file exported from R following digitization and verification, served as the direct input to this pipeline. Each phase built directly on the outputs of the preceding step: WhiteRabbit scan reports informed the Rabbit-in-a-Hat mapping design, the mapping specifications guided ETL development in PostgreSQL, and the loaded OMOP data in PostgreSQL was then validated iteratively using Achilles and DQD before being made available for exploration in ATLAS.

### 2.3 Observational health data sciences and informatics tools used

The OHDSI open-source ecosystem provides a suite of tools that support the standardization, quality assessment, and analysis of observational health data within the OMOP CDM (7, 8). In this study, several OHDSI tools were used across the ETL, validation, and analysis phases.

*WhiteRabbit* was used for source data profiling. It scans the clean dataset to generate descriptive statistics on tables, fields, data types, value distributions, and missingness, enabling a comprehensive understanding of the structure and content of the source data prior to transformation.

*Rabbit-in-a-Hat* was used to design the ETL mapping process. By importing the profiling results generated by WhiteRabbit, Rabbit-in-a-Hat supports visual and collaborative source-to-target mapping between the original dataset and OMOP CDM tables and fields, while documenting transformation logic and relationships.

*Usagi* was employed for vocabulary mapping. It facilitates semi-automated mapping of source clinical terms to standard OMOP vocabularies by leveraging similarity scoring and expert review, ensuring semantic consistency across conditions, measurements, and observations.

*ATHENA* served as the vocabulary management platform. It was used to access, download, and manage standardized OMOP vocabularies required for concept mapping and to ensure alignment with the OMOP CDM Version 5.4 specifications.

*The Data Quality Dashboard (DQD)* was used to evaluate the conformance, completeness, and plausibility of the transformed data. DQD executes a comprehensive set of automated data quality checks against OMOP CDM rules, allowing systematic identification and resolution of data quality issues.

*Achilles* was applied for post-load data characterization and quality assessment.

*ATLAS* was configured as the analytical interface for the OMOP CDM database. It enables interactive data exploration, characterization, and visualization, supporting downstream analytical workflows and clinical research use cases.

#### 2.3.1 Source data profiling with whiteRabbit

WhiteRabbit was used to systematically scan and profile the source data, that is, the cleaned dataset, which was uploaded into WhiteRabbit. It performed a comprehensive summary of the source TB database, examining table structures, field characteristics, data distributions, and value frequencies. The tool generated detailed scan reports (Table 2) that documented essential metadata, including table names, column names, data types, and frequency counts. The WhiteRabbit scan report provided the technical foundation for subsequent mapping activities by documenting all source data elements and characteristics. Additionally, it facilitated data quality assessment at the source level, identifying potential issues such as missing values and unexpected value ranges that required attention during the transformation process. It also served as documentation for data governance, creating a permanent record of the source data structure that supports reproducibility and transparency in the transformation process, aligning with the data quality framework principles for improving healthcare data networks (18).

**Table 2:** Overview of TB dataset scan report.

Table	Description	Type	Fraction empty	*N* unique values	Fraction unique
TB_DGH 2009-2022.csv	uniqueid	VARCHAR	0.0%	2,426	100.0%
TB_DGH 2009-2022.csv	age	INT	0.0%	73	3.0%
TB_DGH 2009-2022.csv	sex	VARCHAR	0.0%	2	0.1%
TB_DGH 2009-2022.csv	year_of_treatment_start	INT	0.0%	14	0.6%
TB_DGH 2009-2022.csv	location	VARCHAR	54.5%	6	0.2%
TB_DGH 2009-2022.csv	TB_v2_localisation	VARCHAR	0.0%	3	0.1%
TB_DGH 2009-2022.csv	clinical_category	VARCHAR	0.0%	5	0.2%
TB_DGH 2009-2022.csv	occupation	VARCHAR	39.3%	5	0.2%
TB_DGH 2009-2022.csv	marital_status	VARCHAR	71.5%	5	0.2%
TB_DGH 2009-2022.csv	history_of_tuberculosis	VARCHAR	71.5%	3	0.1%
TB_DGH 2009-2022.csv	HIV_status	VARCHAR	0.0%	4	0.2%
TB_DGH 2009-2022.csv	diabetes	VARCHAR	0.0%	2	0.1%
TB_DGH 2009-2022.csv	chronic_kidney_disease	VARCHAR	0.0%	3	0.1%
TB_DGH 2009-2022.csv	ARV_treatment	VARCHAR	32.6%	4	0.2%
TB_DGH 2009-2022.csv	tobacco_consumption	VARCHAR	0.0%	3	0.1%
TB_DGH 2009-2022.csv	alcohol_consumption	VARCHAR	0.0%	2	0.1%
TB_DGH 2009-2022.csv	nocturnal_excessive_sweating	VARCHAR	23.7%	3	0.1%
TB_DGH 2009-2022.csv	anorexia	VARCHAR	23.7%	3	0.1%
TB_DGH 2009-2022.csv	weight_loss	VARCHAR	23.8%	3	0.1%
TB_DGH 2009-2022.csv	asthenia	VARCHAR	23.9%	3	0.1%
TB_DGH 2009-2022.csv	fever	VARCHAR	23.7%	3	0.1%
TB_DGH 2009-2022.csv	cough	VARCHAR	23.7%	3	0.1%
TB_DGH 2009-2022.csv	expectoration	VARCHAR	23.8%	3	0.1%
TB_DGH 2009-2022.csv	hemoptysis	VARCHAR	23.8%	3	0.1%
TB_DGH 2009-2022.csv	dyspnea	VARCHAR	23.8%	3	0.1%
TB_DGH 2009-2022.csv	chest_pain	VARCHAR	23.9%	3	0.1%
TB_DGH 2009-2022.csv	current_weight	INT	92.0%	52	2.1%
TB_DGH 2009-2022.csv	leukocytes	REAL	58.2%	380	15.7%
TB_DGH 2009-2022.csv	neutrophils	REAL	79.3%	297	12.2%
TB_DGH 2009-2022.csv	lymphocytes	REAL	79.7%	271	11.2%
TB_DGH 2009-2022.csv	eosinophils	REAL	85.3%	79	3.3%
TB_DGH 2009-2022.csv	basophils	INT	87.9%	18	0.7%
TB_DGH 2009-2022.csv	hemoglobin	REAL	58.4%	187	7.7%

The output of WhiteRabbit, a scan report file (.xlsx), was saved and directly imported into Rabbit-in-a-Hat in the subsequent step to initiate the visual ETL mapping design.

#### 2.3.2 Mapping with Rabbit-in-a-Hat

**Figure 2:**
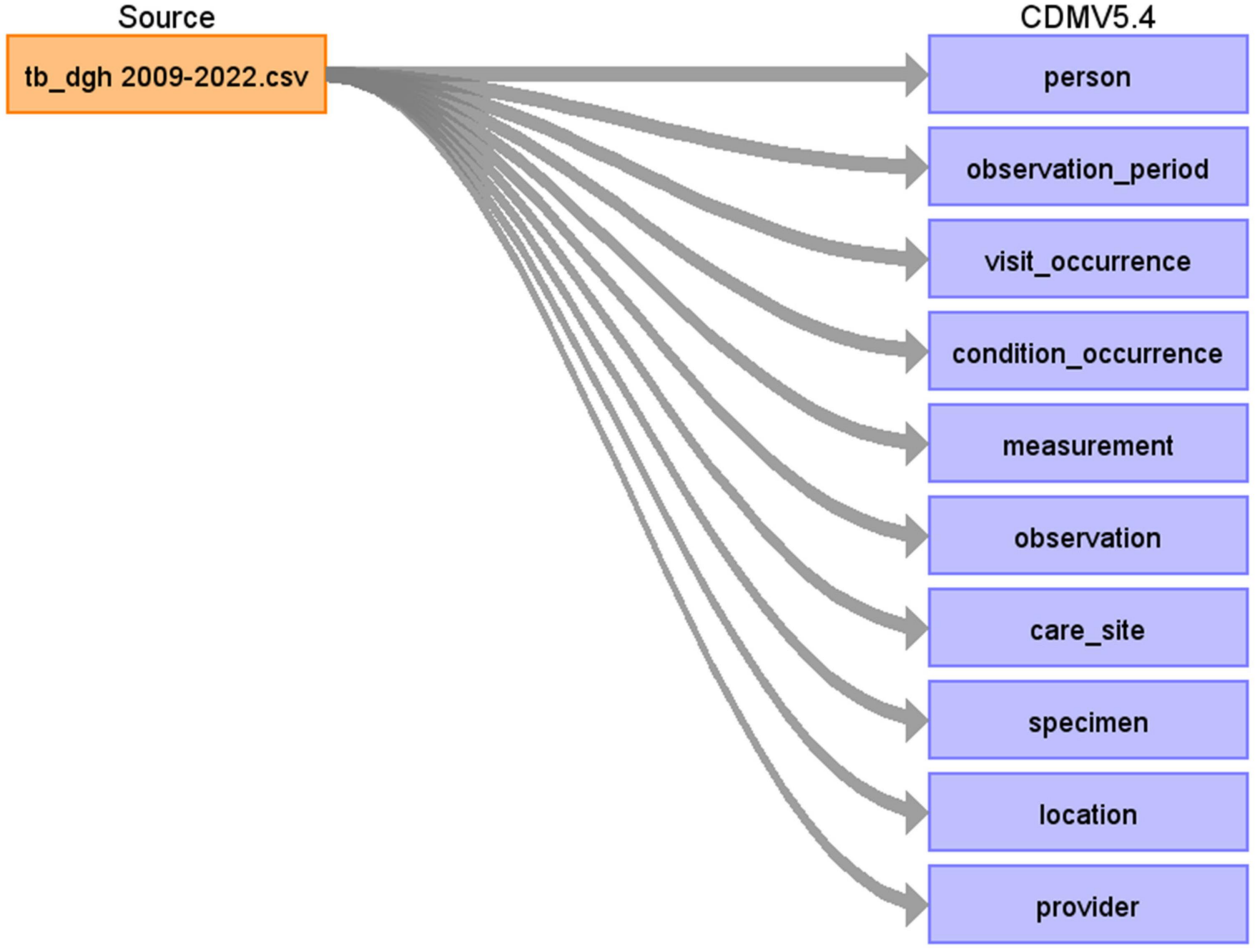
Mapping specifications created for 10 OMOP CDM tables based on the TB dataset.

Following source data profiling, the ETL design was carried out using Rabbit-in-a-Hat. Within the tool, detailed mapping specifications were developed for 10 OMOP CDM tables. These included the *person* table, where patient attributes such as uniqueid, age, and sex were mapped; *observation_period*; *visit_occurrence*; and *condition_occurrence*, which captured current and past medical conditions such as cough, fever, and dyspnea. The *measurement* table contained laboratory and clinical values such as weight, neutrophils, eosinophils, and creatinine, while the *observation* table included non-clinical information such as occupation, marital status, and anorexia. Additional mappings were created for *care_site*, *specimen*, *location*, and *provider* tables, as illustrated in Figure 2.

**Figure 3:**
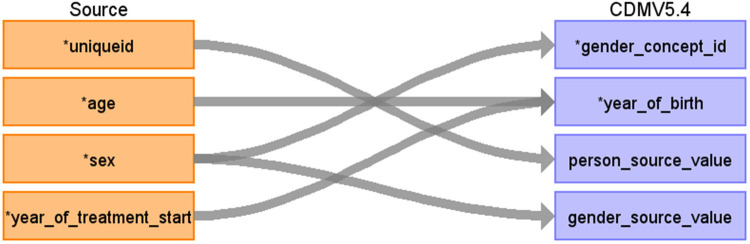
Detailed mapping of the person table based on the TB dataset.

Figure 3 provides a detailed example of the mapping for the *person* table, where *uniqueid* was mapped to *person_source_value*, *age* to *year_of_birth*, *sex* to both *gender_concept_id* and *gender_source_value*, and *year_of_treatment_start* to *year_of_birth*. This structured process was applied similarly across the remaining nine tables to ensure consistency and transparency in the transformation logic.

Rabbit-in-a-Hat also facilitated the generation of a mapping specification document that included visual diagrams serving as reference materials throughout the implementation process, as shown in Figures 2, 3. The visual representation of mappings enabled rapid identification of potential gaps or inconsistencies in the mapping logic, supporting iterative refinement of the transformation specifications before proceeding to implementation.

Table 3 presents an extract of the ETL mapping specification for the *person* table, showing the code-level mappings applied during the transformation. The same approach was replicated across the remaining nine tables, and the finalized ETL specification document was subsequently handed over for actual ETL development.

**Table 3:** ETL specification document extracted from a person table.

Destination field	Source field	Comment
person_id		Autogenerated
gender_concept_id	sex	This field is meant to capture the biological sex at birth of the Person.
		Male = 8,507
		Female = 8,532
		This field should not be used to study gender identity issues.
		Data type: integer
year_of_birth	age	Subtract age from the year of treatment start.
	year_of_treatment_start	
race_concept_id		Indicate concept ID for African race (38003600—African)
ethnicity_concept_id		Indicate that all our patients are not Hispanic or Latino (38003564—Not Hispanic or Latino)
location_id		Foreign key obtained from the location Table.

During the mapping process, several challenges were encountered, particularly in mapping terms such as dark_yellow, transfer out, reprocessing, osteoarticular, and ADP puncture, which lacked corresponding concept IDs in ATHENA and Usagi. However, other terms, including pulmonary TB, extrapulmonary TB, male, female, anorexia, fever, neutrophils, and eosinophils, were successfully mapped without difficulty.

#### 2.3.3 Terminology mapping with Usagi

Usagi was employed to support the mapping of local codes and free-text terms to standardized OMOP concept identifiers. This terminology mapping process covered multiple categories of clinical concepts relevant to TB. For diagnostic terminology, local diagnosis terms were mapped to SNOMED concepts representing different presentations of TB and related symptoms, including pulmonary and extrapulmonary TB, fever, cough, chest pain, dyspnea, and other TB-associated conditions.

Laboratory concepts were also mapped, covering bacteriological investigations such as sputum smear microscopy, culture, and molecular assays, as well as biochemical and hematological parameters used in TB diagnosis and treatment monitoring, including hepatitis serology and acid-fast bacilli testing.

**Figure 4:**
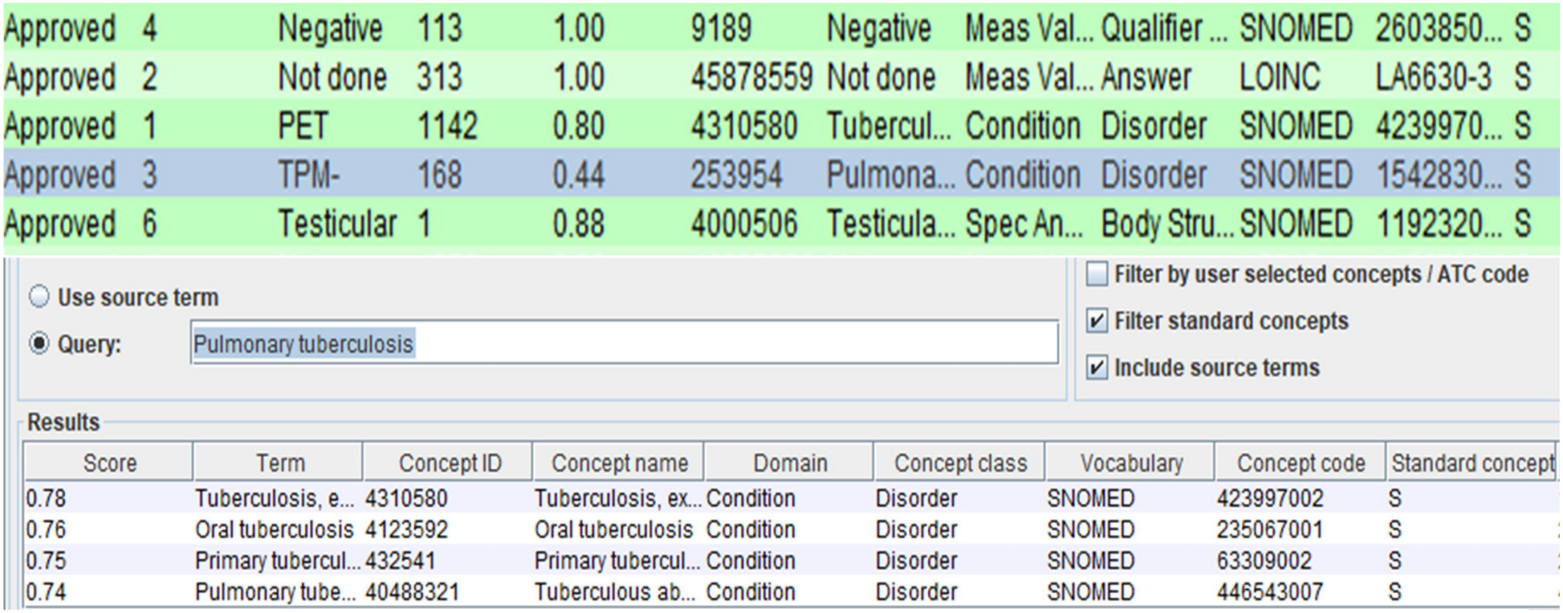
Terminology mapping with Usagi.

For instance, the term TPM- (pulmonary tuberculosis) highlighted in Figure 4 and the full list is included as a supplementary file, generating several candidate concepts from the standard vocabulary, including concept ID: 253954 for pulmonary tuberculosis, concept ID: 4310580 for tuberculosis (extrapulmonary), and concept ID: 40488321 for pulmonary tuberculosis abscess, as shown in Figure 4. Based on the clinical context and the source data, TPM- in the patient records referred to the localization of tuberculosis in the patient's body.

Candidate concepts were evaluated for semantic fit and clinical relevance. Terms with insufficient specificity, such as pulmonary tuberculosis abscess, were excluded to preserve the original clinical concept of the data. Hence, the variable was mapped to concept ID 253954, which refers to pulmonary tuberculosis and is mapped as a condition, as highlighted in Figure 4, since it best represented the source term in a clinically accurate and standardized way. This process was repeated for all the variables in our dataset. The mapping was reviewed by DGH clinicians and subsequently by the Data Science Without Borders Technical team.

Furthermore, during the mapping phase, issues such as incorrect concept placement were encountered. For example, *anorexia* was initially mapped to the *condition_occurrence* table rather than the observation table. This error was later identified during visualization in ATLAS, prompting a revision and re-mapping to ensure accurate classification. Such challenges reflect the complexities noted by Ahmadi et al. (16) in adapting the OMOP CDM for rare diseases, where highly specific clinical terminology often demands careful interpretation to ensure appropriate standardization.

All mapping decisions were recorded in Usagi output files, ensuring a complete and auditable history of terminology transformations. This documentation provides transparency, supports quality assurance, and facilitates future updates. An example of this is illustrated in Figure 4.

#### 2.3.4 Vocabulary management with ATHENA

Through ATHENA's web interface, we identified and downloaded the complete set of vocabulary Version 5 necessary for the TB data transformation, including SNOMED for clinical conditions and findings, LOINC for laboratory tests and measurements, and additional supporting vocabularies such as relationship definitions and domain classifications.

#### 2.3.5 Database infrastructure with PostgreSQL

The database infrastructure for our OMOP CDM implementation was established using PostgreSQL, chosen for its open-source nature, robustness, and strong compatibility with OHDSI tools and workflows. Following OHDSI conventions, we created a PostgreSQL database named TB_DGH with the appropriate schema configurations, as shown in Figure 1, to host both the OMOP CDM data tables and the standardized vocabulary tables from ATHENA. The database structure strictly adhered to the OMOP CDM Version 5.4 specification, ensuring compatibility with OHDSI analytical tools.

Database setup procedures included creating all required OMOP CDM tables with appropriate field definitions and data types. Vocabulary tables were populated with the standard vocabulary downloaded from ATHENA, including the *concept*, *concept_relationship*, *concept_ancestor*, *concept_class, domain*, and related vocabulary management tables, which are essential for supporting standardized terminology operations within the CDM. The next step involved developing SQL scripts and loading the dataset into the respective OMOP CDM tables in PostgreSQL.

Specifically, custom SQL scripts were developed to map each field from the cleaned CSV, previously structured in R, into the corresponding OMOP CDM table columns, applying the concept mappings finalized in Usagi. The CSV file was loaded into a staging schema in PostgreSQL, and the ETL scripts then transformed and moved records from the staging schema into the OMOP CDM schema.

#### 2.3.6 Data quality assessment with achilles and data quality dashboard

After completing the ETL process into the OMOP CDM database, a comprehensive data quality assessment was carried out using the OHDSI Achilles and DQD. The Achilles and DQD are open-source R libraries supported in OMOP CDM Versions 5.4, 5.3, and 5.2, and they evaluate OMOP CDM implementations by running an extensive set of automated checks aligned with the data quality framework proposed by Kahn et al. (17), encompassing key dimensions such as plausibility, conformance, and completeness (13). It also supports multiple relational database management systems (RDBMS), such as PostgreSQL, Microsoft SQL Server, and Amazon Redshift, which are popular for cloud deployments using the OHDSI or cloud architecture.

The TB dataset underwent multiple quality checks through the Achilles and DQD. Plausibility checks assessed whether data values fell within realistic clinical ranges, detecting potential quality issues such as invalid event dates, implausible laboratory measurements, or inconsistent temporal sequences between clinical events. Conformance assessments ensured that data adhered to OMOP CDM structural and semantic requirements, including correct use of foreign keys, valid standard concept identifiers, appropriate domain assignments, and proper differentiation between standard and source concept fields. Completeness evaluations examined the presence of mandatory data elements, identifying missing values or sparsely populated fields that could indicate ETL issues or limitations inherent to the source dataset. These checks were guided by predefined rule definitions documented in the Achilles and DQD specifications.

The Achilles and DQD produced detailed reports presenting results at both summary and table levels, which enabled both systematic review and prioritisation of remediation issues. Following the initial assessment, an iterative data quality improvement process was undertaken. The identified issues were resolved through adjustments to the ETL script, corrections to the source data, and refinements to the concept mapping. The DQD was then re-executed to validate improvements and confirm that quality indicators had improved as expected. This continuous improvement approach aligns with the best practices described by Marteau et al. (11), demonstrating the value of DQD in enhancing the reliability of large healthcare research data repositories.

#### 2.3.7 Analytical platform configuration with ATLAS

The final phase of our implementation involved configuring ATLAS, a low-code OHDSI web-based analytical platform that provides comprehensive functionality for exploring OMOP CDM data and visualizing results (21). ATLAS was deployed and configured to connect to our PostgreSQL OMOP database. ATLAS provided access to the full suite of OHDSI analytical capabilities for our TB dataset. The platform configuration included setting up appropriate user authentication, defining data source connections, and configuring analytical parameters tailored to our research objectives and institutional requirements.

## 3 Results

This section presents the results of harmonizing the TB dataset into the OMOP CDM.

### 3.1 Data quality dashboard output

**Figure 5:**
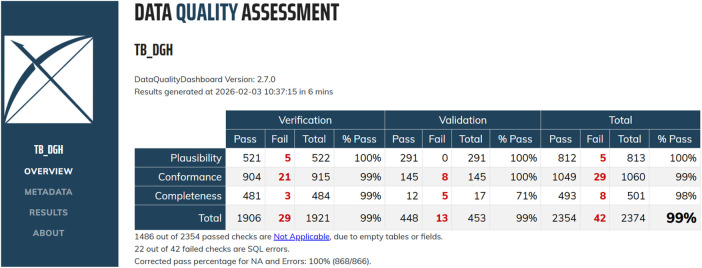
Data quality dashboard for the harmonized TB dataset.

After the ETL process, data quality was evaluated using the Achilles and DQD, and the results are summarized in Figure 5. The assessment demonstrated high plausibility, conformance, and completeness, with the dashboard reporting an overall quality score of 99%.

A total of 2,374 data quality checks were generated (1,921 verification checks and 453 validation checks), of which 2,354 were reported as passed and 42 as failed. A substantial proportion of the passed checks (1,486) were classified as not applicable. These checks relate primarily to OMOP CDM tables or fields that are empty or not expected to be populated in this tuberculosis-specific dataset. NA checks are counted as passed in the dashboard output but are excluded from the corrected pass rate calculation.

In addition, 22 of the 42 failed checks were due to SQL execution errors rather than true data quality violations. When excluding both NA checks and SQL execution errors, all remaining applicable checks passed (868/868 = 100%). This indicates that the ETL implementation is structurally aligned with OMOP CDM requirements and that only a small number of issues require further review.

### 3.2 ATLAS output

**Figure 6:**
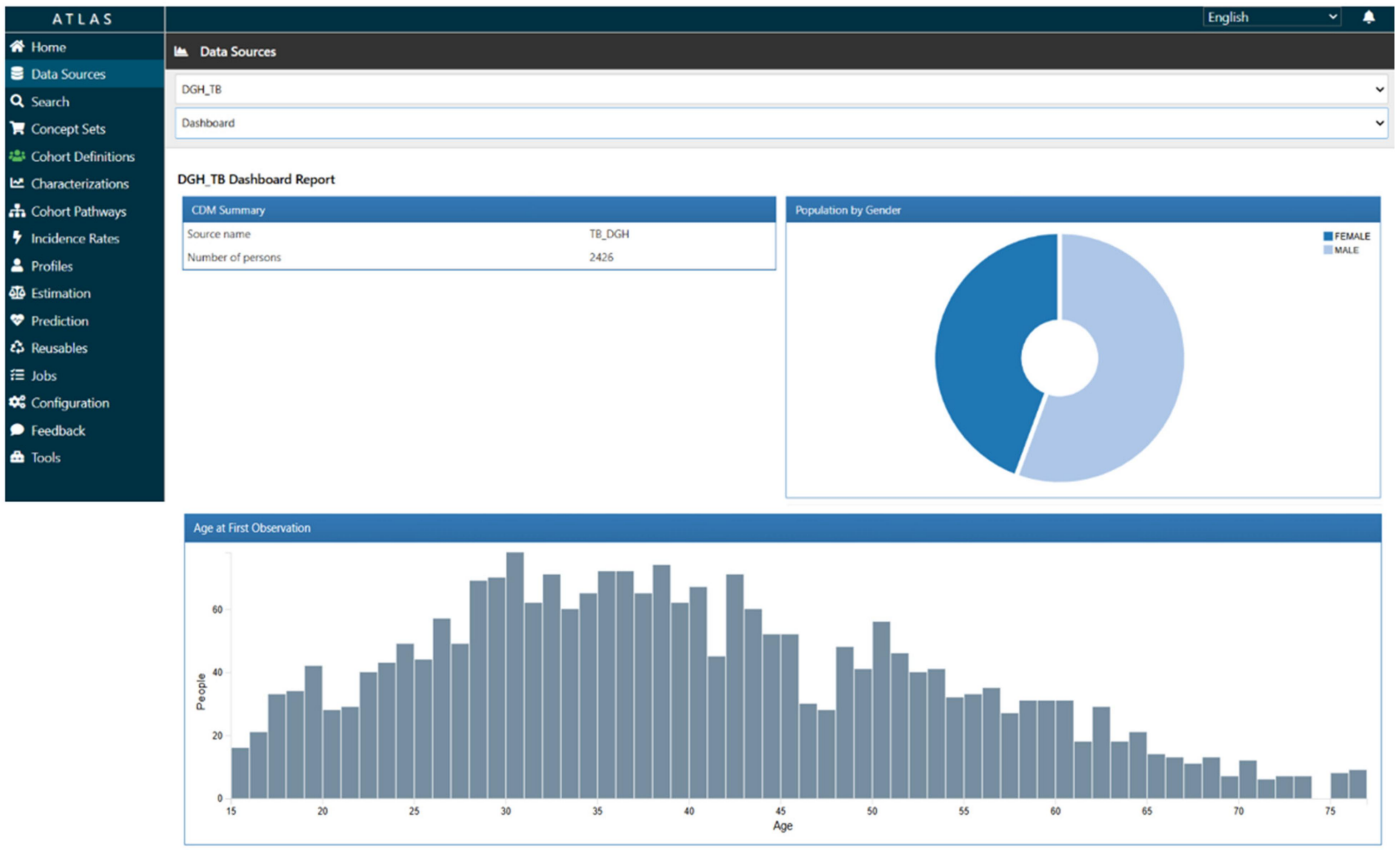
ATLAS dashboard report for the harmonized TB dataset. Screenshot from ATLAS (https://atlas-demo.ohdsi.org/), licensed under Apache License 2.0.

Figure 6 presents the ATLAS dashboard reports and visualizations generated from the harmonized TB dataset. Using the data sources module in ATLAS, we explored dataset characteristics summarized by Achilles. The harmonized cohort consisted of 2,426 individuals, of whom 55.6% (1,349) were male, and 44.4% (1,077) female. Participants' ages ranged from 15 to 95 years, with the most significant proportion clustered between 30 and 40 years.

**Figure 7:**
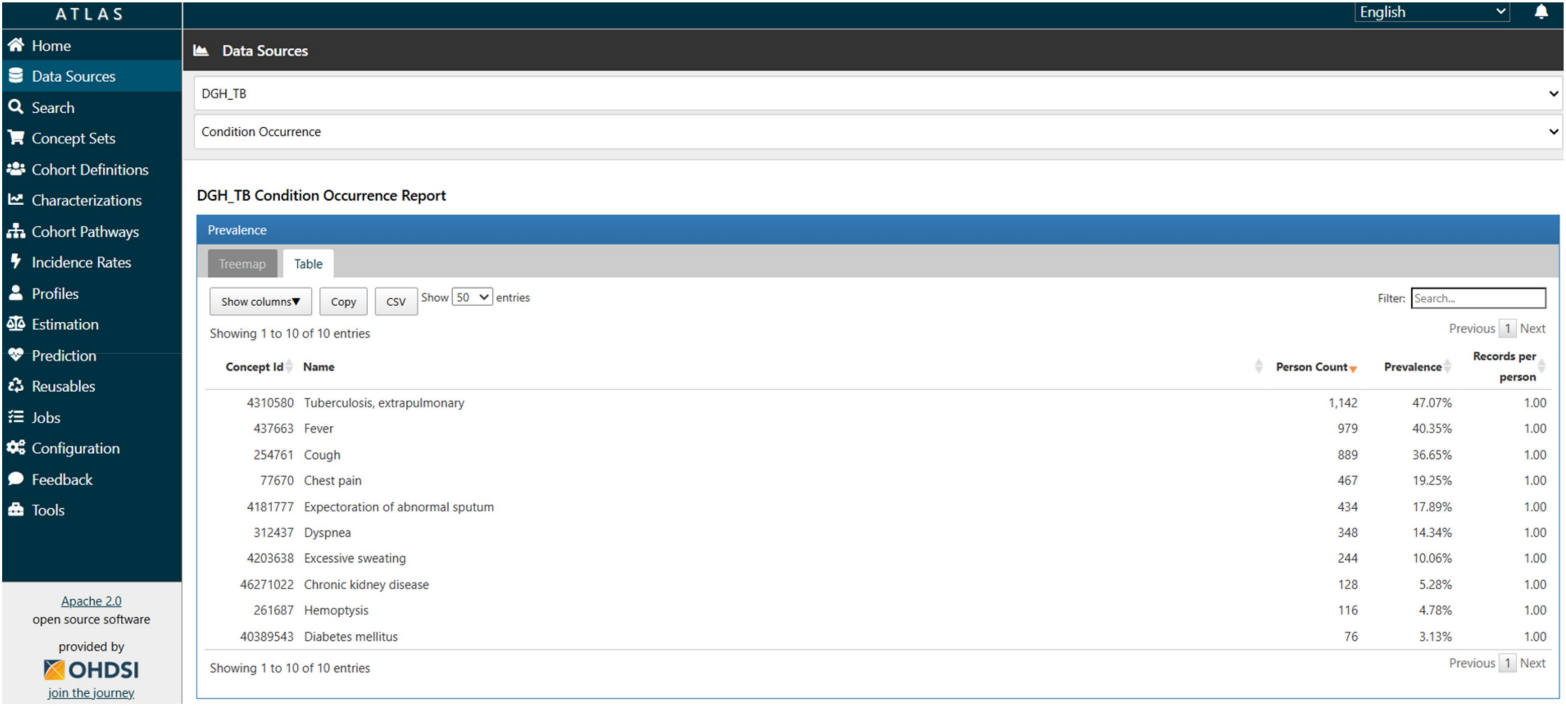
ATLAS condition occurrence report for the harmonized TB dataset. Screenshot from ATLAS (https://atlas-demo.ohdsi.org/), licensed under Apache License 2.0.

Figure 7 illustrates the prevalence of key clinical conditions recorded in the dataset. The most frequently observed conditions included extrapulmonary tuberculosis, fever, cough, chest pain, and expectoration of abnormal sputum. In this retrospective cross-sectional dataset, each individual contributed a single condition record, reflecting the structure and scope of the original clinical data.

**Figure 8:**
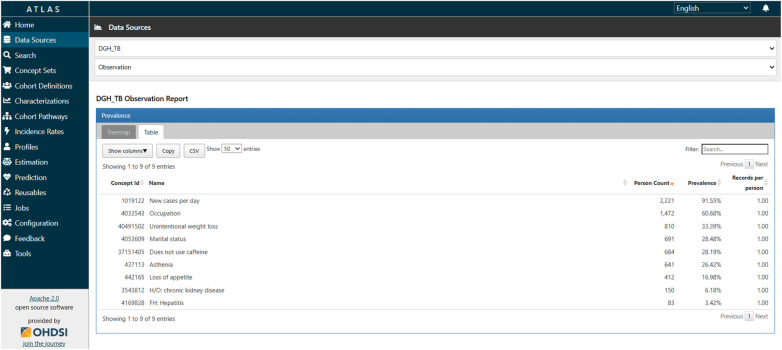
ATLAS condition observation report for the harmonized TB dataset. Screenshot from ATLAS (https://atlas-demo.ohdsi.org/), licensed under Apache License 2.0.

Figure 8 presents the prevalence of observations recorded in the dataset. In this retrospective cross-sectional study, the most frequent observation was the reporting of new cases per day, followed by occupation, unintentional weight loss, and marital status. As with other components of the dataset, each participant contributed a single observation record, consistent with the structure of the original data collection.

**Figure 9:**
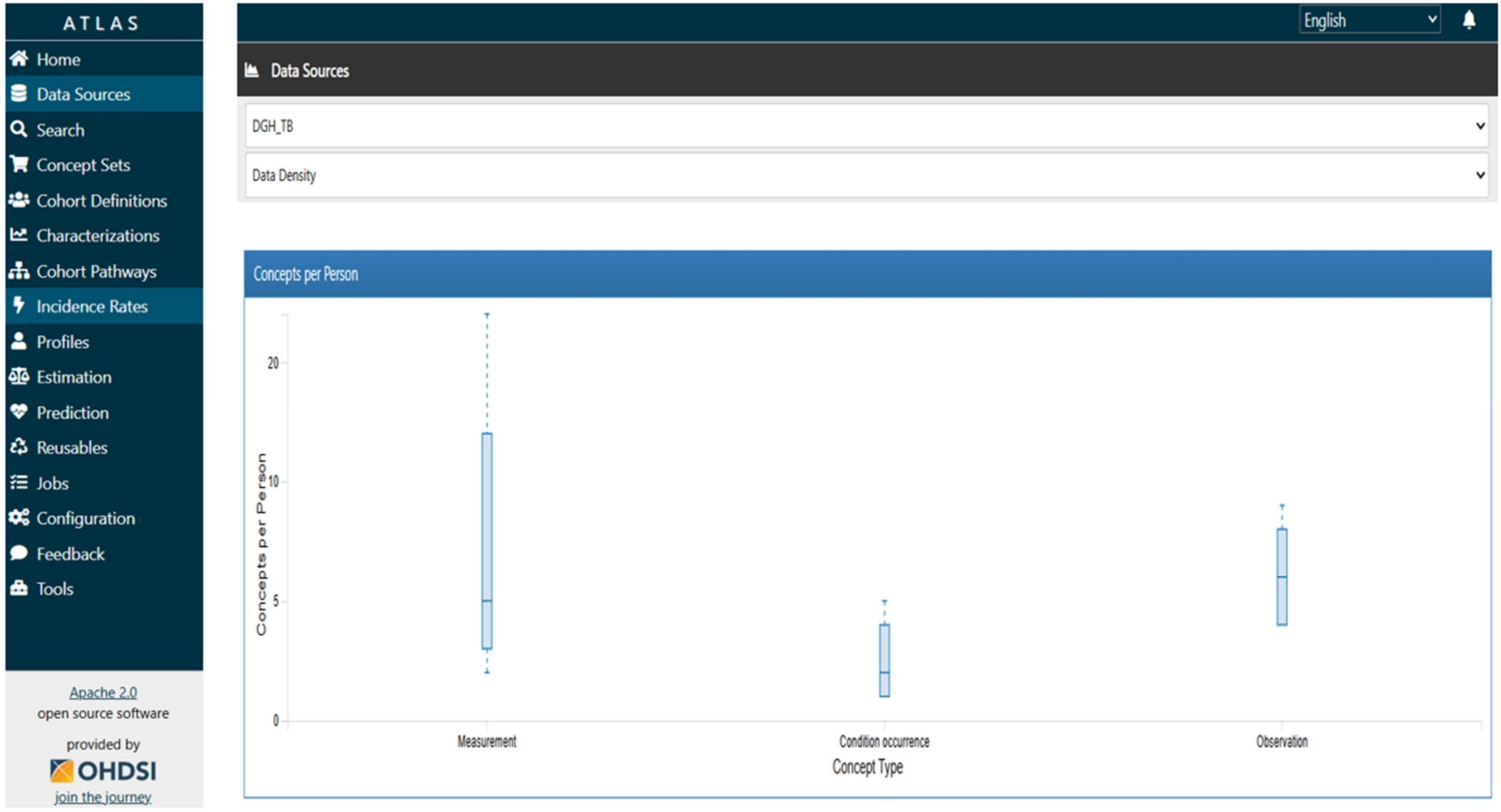
ATLAS data density plot on measurement, condition occurrence, and observation for the harmonized TB dataset. Screenshot from ATLAS (https://atlas-demo.ohdsi.org/), licensed under Apache License 2.0.

The median number of reported measurements per individual was 5, compared with 2 for conditions and 6 for observations. However, variability was observed across the cohort, with some individuals contributing up to 22 measurements, 9 conditions, and 12 observations, as illustrated in Figure 9.

## 4 Discussion

This study demonstrates the successful harmonization of a hospital-based TB dataset from DGH into the OMOP CDM, confirming that clinical datasets originating from non-standardized paper and digital systems in resource-limited settings can be transformed into internationally interoperable formats. The transformation pipeline employed widely adopted OHDSI tools, enabling structured profiling, mapping, vocabulary alignment, database deployment, and iterative data quality assurance, reflecting best practices for OMOP implementations.

The resulting harmonized dataset achieved a high level of structural integrity, with 2,374 data quality checks executed and a 99% pass rate. The strong performance across plausibility, conformance, and completeness indicates that the ETL process preserved the semantic meaning and usability of the original data while meeting OMOP specifications. Such outcomes align with the experiences of other healthcare systems that have demonstrated the value of the DQD in systematically strengthening research-grade data repositories (11–16, 18, 20, 22).

A key benefit of the harmonization was improved semantic interoperability. Usagi enabled the mapping of locally defined clinical concepts, such as extrapulmonary TB, neutrophils, and cough, to standard SNOMED and LOINC vocabularies, which is essential for meaningful querying, phenotyping, and participation in multi-centre or cross-country studies. However, several source concepts, including dark_yellow, transfer out, reprocessing, osteoarticular, and ADP puncture, could not be mapped because their corresponding standard concept identifiers were absent in ATHENA and Usagi. These unmapped terms were retained as source values, and future work will involve proposing new concept mappings through the OHDSI vocabulary contribution process and periodically updating ATHENA releases to incorporate newly standardized concepts as they become available. These challenges were also observed in the study on rare disease implementations by Ahmadi et al. (16).

The implementation also demonstrated the importance of iterative refinement and highlighted the largely manual nature of several validation steps, which presents a challenge for scalability and shows the need for greater automation. Errors such as the misclassification of anorexia into the wrong domain only became apparent after visual review in ATLAS, which demonstrates that tool-centred validation alone is insufficient, and that human oversight reinforces the OMOP CDM learning curve for new implementers and emphasises that harmonization is not a one-time process but a continual evolution as vocabularies, data structures, and analytical requirements advance.

Several specific challenges inherent to the resource-limited settings of this study require explicit discussion, as they meaningfully shaped the implementation process and distinguish this work from comparable OMOP CDM implementations undertaken in better-resourced settings. First, the primary data source consisted of paper-based clinical records spanning 13 years. The digitization of 2,426 patient records and 82 variables was a substantial manual undertaking, requiring dedicated personnel time and domain expertise. Unlike settings where data already exist in electronic form, even if imperfectly structured, our team had to complete this foundational step before any OMOP tooling could be applied, representing a significant additional barrier in Sub-Saharan African healthcare institutions where paper-based records remain the norm. Second, unreliable internet connectivity at the hospital posed practical difficulties during vocabulary downloads from ATHENA, tool installations, and access to OHDSI community resources and documentation, all of which assume stable broadband access. These interruptions introduced delays and required offline workarounds not documented in standard OHDSI implementation guides. Third, the capacity-building component was substantial: team members required training in ETL design, SQL scripting, R-based quality tools, and OMOP CDM concepts prior to and during implementation. Unlike institutions with established OMOP expertise, this project operated without prior in-house experience, making the learning curve steeper and the timeline longer. Finally, the mapping of locally used clinical terminology, including French-language terms and facility-specific abbreviations such as TPM- and ADP puncture, to international standard vocabularies (SNOMED, LOINC) revealed gaps in current OMOP vocabulary coverage for African clinical contexts. These factors illustrate that while the OHDSI tool suite is technically accessible in resource-limited settings, the surrounding conditions, including data infrastructure, connectivity, human capital, and vocabulary coverage, add layers of complexity that are not captured when the implementation is described solely through its technical outputs.

The ATLAS analytical dashboards confirmed the dataset's readiness for research and secondary analysis. The demographic and clinical summaries produced, such as the predominance of adult patients aged 30–40 and the high prevalence of core TB symptoms (26), were consistent with regional TB epidemiology and provided a foundation for future real-world evidence studies, trend monitoring, treatment outcome assessment, and surveillance initiatives.

Importantly, this study serves as a proof of concept for the feasibility of OMOP CDM deployments in low-resource contexts where electronic data governance and standardization frameworks are still evolving. Lessons learned here can inform national and regional digital health strategies, demonstrating that with appropriate tooling and capacity building, high-quality interoperable datasets can be achieved without proprietary platforms or cost barriers. However, it is important that future implementations in similar contexts are accompanied by realistic planning for the additional effort required. Specifically, institutions considering OMOP CDM adoption in resource-limited settings should budget for the digitization of their data, allocate time for team training across ETL, SQL, and vocabulary management competencies, plan for intermittent connectivity when downloading ATHENA vocabularies and accessing OHDSI tools, and engage with the OHDSI community to advocate for expanded vocabulary coverage of clinical concepts prevalent in SSA. Documenting and sharing these contextual barriers is essential for building a realistic and replicable model of OMOP CDM adoption across the African continent.

## 5 Conclusion

This study successfully demonstrated the feasibility of implementing the OMOP CDM to standardize TB clinical data from a major healthcare institution in Cameroon. By leveraging the OHDSI open-source tool ecosystem, the dataset was successfully profiled, mapped, transformed, and evaluated, resulting in a highly interoperable resource suitable for secondary research use. The strong performance on data quality checks and the clear analytical outputs in ATLAS confirm that the resulting dataset is structurally sound and semantically consistent.

Notably, the implementation highlights that even in contexts where digital health infrastructure is evolving, standards-based harmonization is achievable with appropriate tools, training, and iterative refinement. The work lays a foundation not only for improved TB surveillance and research within the country but also for participation in regional and global studies. Broader adoption of the OMOP CDM in SSA could accelerate data-driven clinical insights, strengthen public health decision-making, and support collaborative evidence generation across health systems.

### 5.1 Recommendations

Based on the findings of this study, the following recommendations are made.

1. There is the need to expand standardized data harmonization beyond a single institution would enable multi-centre studies and more comprehensive national analyses.
2. Linking pharmacy, imaging, microbiology, and treatment follow-up records would enable outcome tracking and comparative effectiveness research.
3. Regular re-execution of the DQD and ATLAS reporting should be institutionalised to maintain data reliability through system upgrades and new data integrations.
4. Continuous training of local data managers, analysts, and clinicians in ETL, terminology management, and OMOP analytical tools will sustain adoption and support wider national standardization.
5. Automating key ETL validation, data quality checks, and domain-mapping workflows is recommended to reduce manual effort, improve reproducibility, and enhance scalability of OMOP CDM implementations.

## Data Availability

The raw data supporting the conclusions of this article will be made available by the authors, without undue reservation.
